# Ecotoxicological hazard of a mixture of glyphosate and aminomethylphosphonic acid to the mussel *Mytilus galloprovincialis* (Lamarck 1819)

**DOI:** 10.1038/s41598-019-50607-0

**Published:** 2019-10-04

**Authors:** Valerio Matozzo, Marco Munari, Luciano Masiero, Livio Finos, Maria Gabriella Marin

**Affiliations:** 1 Department of Biology, University of Padova, Via Ugo Bassi 58/B, 35131 Padova, Italy; 2 Department of Integrative Marine Ecology, Villa Dohrn-Benthic Ecology Center Ischia, Stazione Zoologica Anton Dohrn, Villa Comunale, 80121 Naples, Italy; 3 Department of Developmental Psychology and Socialisation, University of Padova, Via Venezia 8, Padova, Italy

**Keywords:** Animal physiology, Environmental impact

## Abstract

Assessment of the effects of chemical mixtures is a very important objective of the ecotoxicological risk assessment. This study was aimed at evaluating for the first time the effects of a mixture of glyphosate and its main breakdown product aminomethylphosphonic acid (AMPA) on various biomarkers in the mussel *Mytilus galloprovincialis*. Mussels were exposed for 7, 14 and 21 days to either 100 µg/L of glyphosate, 100 µg/L of AMPA or a mixture of both (100 + 100 µg/L). Various haemocyte parameters, such as total haemocyte counts, haemocyte diameter and volume, haemocyte proliferation, haemolymph lactate dehydrogenase activity and haemocyte lysate acid phosphatase activities were measured. In addition, the effects of exposure on the activity of antioxidant enzymes, acetylcholinesterase and glutathione-S-transferase were evaluated in gills and digestive gland from mussels. On the whole, this study demonstrated that the variables considered in the experimental plan, namely treatment, exposure time and their interaction, affect significantly biomarker responses in *M*. *galloprovincialis*. The effects of the mixture were comparable to those of the individual compounds, whereas their synergistic effects were occasionally observed, under the experimental conditions tested at least.

## Introduction

Glyphosate is a broad-spectrum, systemic, non-selective, and post-emergence herbicide applied widely as a plant growth regulator. Indeed, glyphosate affects essential aromatic amino acids synthesis in plants, inhibiting protein synthesis^1,2^. Due to its effectiveness, such compound is an important ingredient of numerous commercial formulations^3^. After application, a remarkable fraction of glyphosate can deposit directly on the ground, where it can be degraded or leached. Microorganisms can degrade glyphosate to aminomethylphosphonic acid (AMPA) or sarcosine^4,5^. Consequently, glyphosate and AMPA can be found together in environment.

Regarding aquatic environments, glyphosate and AMPA are detected at measurable concentrations. In USA, the two compounds occurred in various ecosystems with a maximum level of 700 μg/L^6^. In Argentina, mean concentrations of glyphosate and AMPA of 0.73 μg/L and 0.53 μg/L, respectively, were found in various water bodies^7^. A recent study demonstrated that the levels of glyphosate and AMPA measured in epilithic biofilms can vary seasonally (from 90 to 305 μg/kg for glyphosate and from 50 to 240 μg/kg for AMPA, in fall and spring, respectively) and suggested that herbicide applications can influence markedly environmental concentrations of the compounds; interestingly, protected sites or with poor access did not show the presence of such contaminants^8^. Glyphosate has been detected in 39.7% of Italian surface water samples, with 25.2% of cases exceeding 0.1 μg/L, namely the annual environmental quality standard (SQA-MA) for rivers under current legislation^9^. Regarding seawater, glyphosate (from 28 to 1690 ng/L) and AMPA (from 45 to 4156 ng/L) have been detected in the estuaries of the German coastline. That study demonstrated that glyphosate and AMPA enter the Baltic Sea^10^. Detectable concentrations of glyphosate (from 13.6 to 1377 µg/L) and AMPA (from 2.58 to 140.8 µg/L) were found in Western Pacific seawater samples^11^. Mercurio *et al*.^12^ recorded half-life values of 47 days for glyphosate in seawater at 25 °C in low-light, of 267 days in the dark at 25 °C and of 315 days in the dark at 31 °C, while AMPA was detected under all conditions. That study demonstrated that glyphosate has a moderate persistence in seawater under low light conditions, but its persistence increases markedly in the dark.

Information available in the literature demonstrate that glyphosate (as active ingredient or commercial formulation) and AMPA affect various biological parameters in non-target aquatic organisms, such as fish^13,14^, amphibians^15^, crustacea^16–18^ and molluscs^19–23^. In two distinct experiments, we have recently demonstrated that exposure to glyphosate (10, 100 and 1000 µg/L) and AMPA (1, 10 and 100 µg/L) affected biomarker responses in *Mytilus galloprovincialis*^24,25^, sometimes with similar effects, sometimes with opposing effects.

Most ecotoxicological studies (including ours) have previously been performed to evaluate the effects of individual chemicals on aquatic species. However, aquatic organisms can be exposed to a mixture of different chemicals in the environment. Consequently, in the present study, we evaluated for the first time the impact of a mixture of glyphosate (as active ingredient) and AMPA on various cell and tissue biomarkers of *M*. *galloprovincialis*. Considering that **i**) glyphosate and AMPA can be found together in aquatic environments (including seawater) and that **ii**) they affect individually biomarker responses in mussels (sometimes with contrasting effects)^24,25^, we assessed the effects of realistic concentrations (100 µg/L) of glyphosate and AMPA and of a mixture of both (100 + 100 µg/L) to mussels.

## Materials and Methods

### Experimental design

Specimens of *M*. *galloprovincialis* (about 6–7 cm shell length) were sampled in a licensed area for bivalve culture in the Lagoon of Venice (Italy). Before experiments, bivalves were acclimated in the laboratory for one week in large aquaria with aerated seawater (salinity of 35 ± 1, temperature of 18 ± 0.5 °C) and were fed daily *ad libitum* with microalgae (*Isochrysis galbana*). Only healthy animals were used for the experiments.

Two separate stock solutions (0.1 g/L, in distilled water) of glyphosate and AMPA (Sigma-Aldrich, Milano, Italy) were prepared, whereas exposure concentrations were obtained by diluting the stock solutions in seawater. Bivalves (seventy per concentration) were exposed for 7, 14 and 21 days to 100 µg/L of glyphosate, 100 µg/L of AMPA and a mixture of 100 µg/L of glyphosate + 100 µg/L of AMPA. Such concentrations were selected on the basis of both information on glyphosate and AMPA levels in aquatic ecosystems reported in Introduction and effects on *M*. *galloprovincialis*^24,25^. Analytical verification of exposure concentrations that were tested in this study has already been performed in our previous studies^24,25^. Bivalves (thirty-five per tank) were kept in 35 L glass tanks (two per concentration). Seawater, stock and working solutions and microalgae were renewed every two days.

### Haemolymph and tissue collection

Haemolymph was sampled from the anterior adductor muscle with a 1-mL plastic syringe and stored at 4 °C in Eppendorf tubes. After 7, 14 and 21 days, pools of haemolymph (5 pools, from 4 bivalves each) from the experimental tanks were prepared. Aliquots of haemolymph were immediately used to measure total haemocyte count (THC), haemocyte diameter and volume, haemocyte proliferation and lactate dehydrogenase (LDH) activity, whereas the remaining aliquots were frozen in liquid nitrogen and stored at −80 °C until analyses. Gills and digestive glands (twenty per concentration) were excised and then pooled (5 pools of 4 mussels each). Subsamples of each pool of tissues were frozen in liquid nitrogen and stored at −80 °C until analyses.

### Haemocyte parameters

The total haemocyte count (THC), haemocyte diameter and volume were measured using a Scepter™ 2.0 Automated Cell Counter (Millipore, FL, USA). Haemolymph samples (20 µL) were mixed to 2 mL of Coulter Isoton II diluent. THC values were expressed as the number of haemocytes/mL of haemolymph, whereas cell diameter and volume were expressed in µm and picolitres (pL), respectively.

A kit (*Cell proliferation* Kit II, Roche) was used to evaluate haemocyte proliferation, as described previously^26^. Absorbance values at 450 nm were recorded with a Beckman 730 spectrophotometer. The results were expressed as optical density OD_450_/mL of haemolymph.

Another kit (*Cytotoxicity Detection* Kit, Roche) was used to measure lactate dehydrogenase activity (LDH) in cell-free haemolymph (CFH). To obtain CFH, haemolymph samples (500 µL) were centrifuged at 780 × g for 10 min, and supernatant (=CFH) was then collected for the assay following the manufacturer’s instructions. The results were expressed as OD_490_/mL haemolymph.

The *Acid Phosphatase Assay* kit (Sigma-Aldrich, Milano, Italy) was used to measure acid phosphatase activity in haemocyte lysate (HL) according to the manufacturer’s instructions. To obtain HL, haemolymph samples were centrifuged at 780 × g for 10 min, and haemocytes were resuspended in distilled water, mixed for 15 sec using a vortex mixer and centrifuged at 10,000 × g for 10 min. The supernatant (=HL), was removed for the acid phosphatase assay. The results were expressed as U/mg protein. Protein concentrations in HL were quantified according to Bradford^27^.

### Tissue preparation and enzyme activity measurement

Gills and digestive gland samples were homogenised on ice with an Polytron homogeniser (PT 1200 E) in five volumes of 10 mM Tris-HCl buffer, pH 7.5, containing 0.15 M KCl, 0.5 M sucrose, 1 mM EDTA and protease inhibitor cocktail (Sigma-Aldrich), and centrifuged at 12,000 g for 45 min at 4 °C. Supernatants (SN) were used for analyses.

SOD activity was measured in triplicate using the xanthine oxidase/cytochrome c method^28^. Results were expressed as U/mg protein; one unit of SOD being defined as the amount of sample causing 50% inhibition under the assay conditions. SN protein concentrations were quantified according to Bradford^27^.

CAT activity was determined in triplicate following the method of Aebi^29^. Results were expressed as U/mg protein; one unit of CAT is defined as the amount of enzyme that catalysed the dismutation of 1 μmol of H_2_O_2_/min. SN protein concentrations were also measured^27^.

Gill acetylcholinesterase (AChE) activity was measured following the method of Ellman *et al*.^30^. The results were expressed as nmol/min/mg of protein. The protein concentration in SN was quantified according to Bradford^27^.

Glutathione S-transferase (GST) activity was measured in digestive gland SN according to the method described in Habig *et al*.^31^ and results were expressed as nmol/min/mg protein.

### Statistical analysis

The normal distribution of data (Shapiro-Wilk’s test) and the homogeneity of the variances (Bartlett’s test) were assessed. The whole data set (“all parameters” in Table 1) was statistically analysed using the Permutational multivariate Analyses of Variance (PERMANOVA, with 9999 permutations), in order to detect significant effects of treatment, exposure time and the interaction between treatment and time. The variables “treatment”, “exposure time” and their interaction were considered as fixed factors. Data from each biomarker were also analysed with PERMANOVA to determine significant effects of the variables considered on haemocytes/haemolymph and soft tissues parameters. In addition, the non-parametric post hoc test (Mann-Whitney *U* test) was used for pairwise comparisons of results from control and treated groups at each sampling time. All results were expressed as the mean ± standard deviation (SD). The software packages Statistica 13.4 (TIBCO Software Inc.) and PRIMER 6 PERMANOVA Plus (PRIMER-E Ltd, Plymouth, UK) were used for the statistical analyses. Lastly, a canonical correlation analysis (CCA) was performed, including exposure concentrations, exposure duration, and the measured cellular and biochemical parameters as variables. The software package R^32^ with the CCA package^33^ were used.

**Table 1:** PERMANOVA analysis results. Pseudo-*F* and p values are reported. Statistically significant effects of the variables “treatment”, “exposure time” and “treatment*time interaction” are indicated in bold.

Variables	All parameters	All haemocyte parameters	All soft tissue parameters	All gill parameters	All digestive gland parameters	Haemocyte parameters						Gills			Digestive gland		
						THC	Cell diameter	Cell volume	Cell proliferation	LDH	Acid phosphatase	SOD	CAT	AChE	SOD	CAT	GST
Treatment	F_(3,48)_ = 2.096 p = **0.040**	F_(3,48)_ = 1.806 p = 0.133	F_(3,48)_ = 2.181 p = **0.040**	F_(3,48)_ = 1.100 p = 0.350	F_(3,48)_ = 2.757 p = **0.040**	F_(3,48)_ = 2.048 p = 0.119	F_(3,48)_ = 1.088 p = 0.367	F_(3,48)_ = 0.962 p = 0.418	F_(3,48)_ = 0.884 p = 0.453	F_(3,48)_ = 1.519 p = 0.228	F_(3,48)_ = 0.220 p = 0.880	F_(3,48)_ = 0.936 p = 0.435	F_(3,48)_ = 0.289 p = 0.836	F_(3,48)_ = 1.109 p = 0.360	F_(3,48)_ = 1.852 p = 0.145	F_(3,48)_ = 0.002 p = **0.002**	F_(3,48)_ = 0.283 p = 0.839
Time	F_(2,48)_ = 15.256 p = **0.000**	F_(2,48)_ = 43.531 p = **0.000**	F_(2,48)_ = 6.965 p = **0.000**	F_(2,48)_ = 18.505 p = **0.000**	F_(2,48)_ = 0.459 p = 0.455	F_(2,48)_ = 52.738 p = **0.000**	F_(2,48)_ = 5.956 p = **0.006**	F_(2,48)_ = 6.351 p = **0.003**	F_(2,48)_ = 4.458 p = **0.014**	F_(2,48)_ = 8.306 p = **0.000**	F_(2,48)_ = 4.749 p = **0.013**	F_(2,48)_ = 0.281 p = 0.753	F_(2,48)_ = 14.871 p = **0.000**	F_(2,48)_ = 16.457 p = **0.000**	F_(2,48)_ = 1.334 p = 0.277	F_(2,48)_ = 0.80 p = 0.070	F_(2,48)_ = 5.142 p = **0.008**
Treatment X Time	F_(6,48)_ = 0.476 p = **0.000**	F_(6,48)_ = 3.181 p = **0.000**	F_(6,48)_ = 1.190 p = 0.289	F_(6,48)_ = 2.199 p = **0.035**	F_(6,48)_ = 0.653 p = 0.702	F_(6,48)_ = 3.9373 p = **0.003**	F_(6,48)_ = 0.357 p = 0.900	F_(6,48)_ = 0.149 p = 0.990	F_(6,48)_ = 3.386 p = **0.008**	F_(6,48)_ = 3.337 p = **0.008**	F_(6,48)_ = 0.708 p = 0.642	F_(6,48)_ = 1.078 p = 0.384	F_(6,48)_ = 1.432 p = 0.223	F_(6,48)_ = 2.062 p = **0.040**	F_(6,48)_ = 0.974 p = 0.451	F_(6,48)_ = 0.758 p = 0.758	F_(6,48)_ = 0.808 p = 0.573

## Results

Statistical analysis indicated that the three variables of the experimental plan (treatment, exposure time and treatment*time interaction) had significant effects on all the biological parameters measured in mussels (Table 1). Regarding the results of post-hoc test at each tissue sampling time, asterisks in the figures denote the significant differences in biomarker responses that were due to the variables “treatment” and “treatment*time interaction” between control and exposed mussels.

### Cell parameters

**Figure 1:**
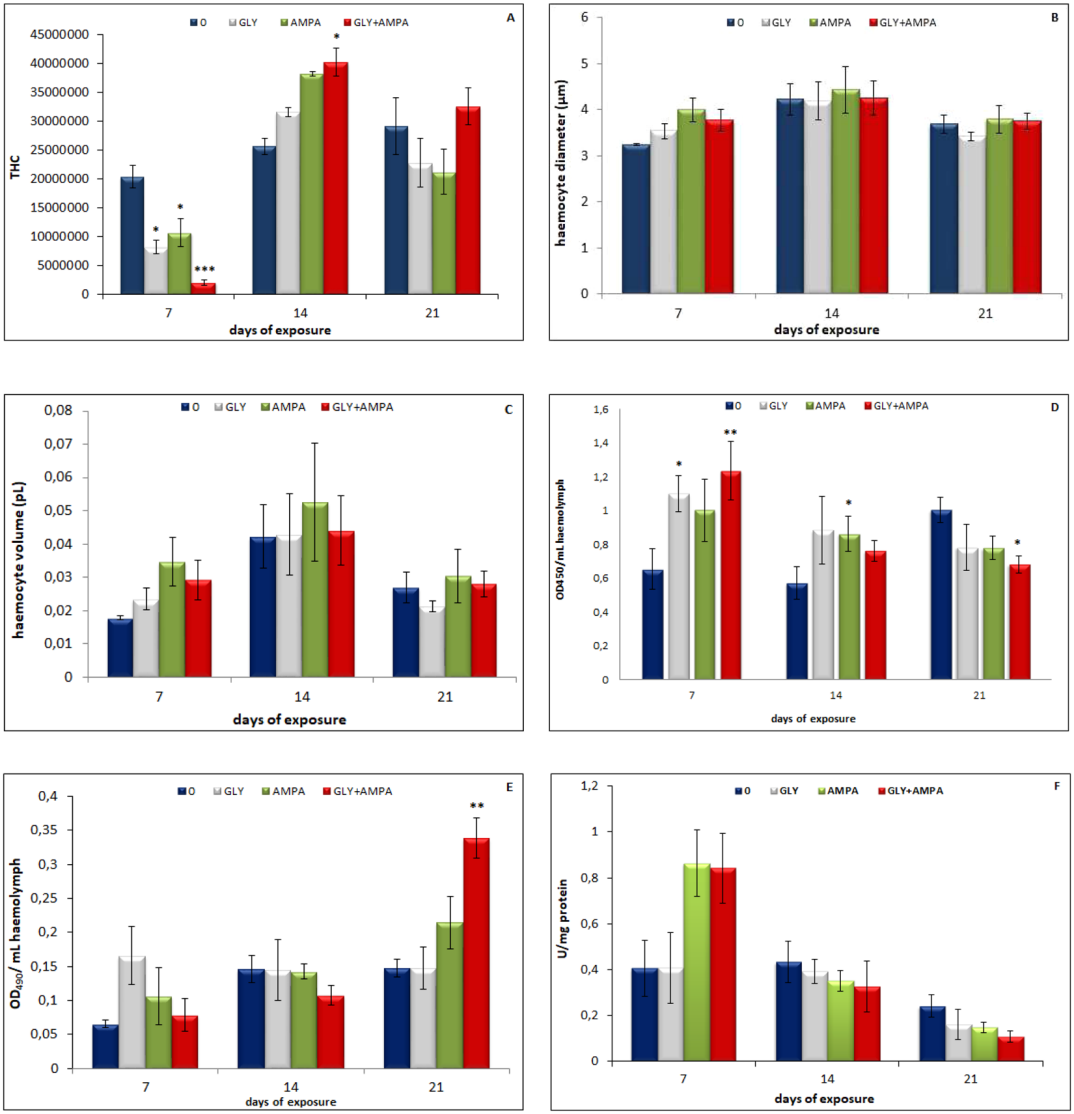
THC, expressed as the number of haemocytes/mL of haemolymph (**A**), haemocyte diameter, expressed in µm (**B**), haemocyte volume, expressed in pL (**C**), haemocyte proliferation, expressed as OD/mL haemolymph (**D**), cell-free haemolymph lactate dehydrogenase activity, expressed as OD/mL haemolymph (**E**), and haemocyte lysate acid phosphatase activity, expressed as U/mg protein (**F**) of *M*. *galloprovincialis* exposed to glyphosate (GLY), AMPA and GLY + AMPA. The values are mean + SD (n = 5). The asterisks indicate significant differences in comparison with controls: *p < 0.05, **p < 0.01, ***p < 0.001.

The variables “exposure time” and “treatment*time interaction” significantly (p < 0.001) influenced haemocyte parameters. In particular, exposure time affected significantly all the haemocyte parameters measured, whereas the variable “treatment*time interaction” influenced significantly THC (p < 0.01), cell proliferation (p < 0.01) and LDH activity (p < 0.01) (Table 1). Regarding pairwise comparisons, THC was shown to decrease significantly in mussels exposed for 7 days to glyphosate (p < 0.05), AMPA (p < 0.05) and the mixture (p < 0.001), whereas a significant (p < 0.05) increase in THC was recorded in mussels exposed for 14 days to the mixture (Fig. 1A).

Haemocyte diameter and volume were affected significantly by the variable “exposure time” only (Table 1; Fig. 1B,C).

Haemocyte proliferation resulted influenced significantly by both the variables “exposure time” (p < 0.05) and “treatment*time interaction” (p < 0.01) (Table 1). As for pairwise comparisons, haemocyte proliferation was shown to increase significantly in animals exposed for 7 days to glyphosate (p < 0.05) and the mixture (p < 0.01), in those treated for 14 days with AMPA (p < 0.05), and decreased significantly (p < 0.05) in mussels exposed for 21 days to the mixture, in comparison with controls (Fig. 1D).

Like haemocyte proliferation, LDH activity was affected significantly by “exposure time” (p < 0.001) and “treatment*time interaction” (p < 0.01) (Table 1). In particular, the exposure for 21 days to the mixture enhanced significantly (p < 0.01) LDH activity, compared to control group (Fig. 1E).

Although acid phosphatase activity increased in HL of mussels exposed for 7 days to both AMPA and the mixture, and decreased in those treated for 14 and 21 days, pairwise comparisons did not reveal significant differences compared with the controls (Fig. 1F).

### Gill and digestive gland enzyme activities

Considering all the parameters measured in soft tissues, namely gills and digestive gland, PERMANOVA analysis indicated that the variables “treatment” and “exposure time” affected significantly (p < 0.05 and p < 0.001, respectively) enzyme activities (Table 1).

**Figure 2:**
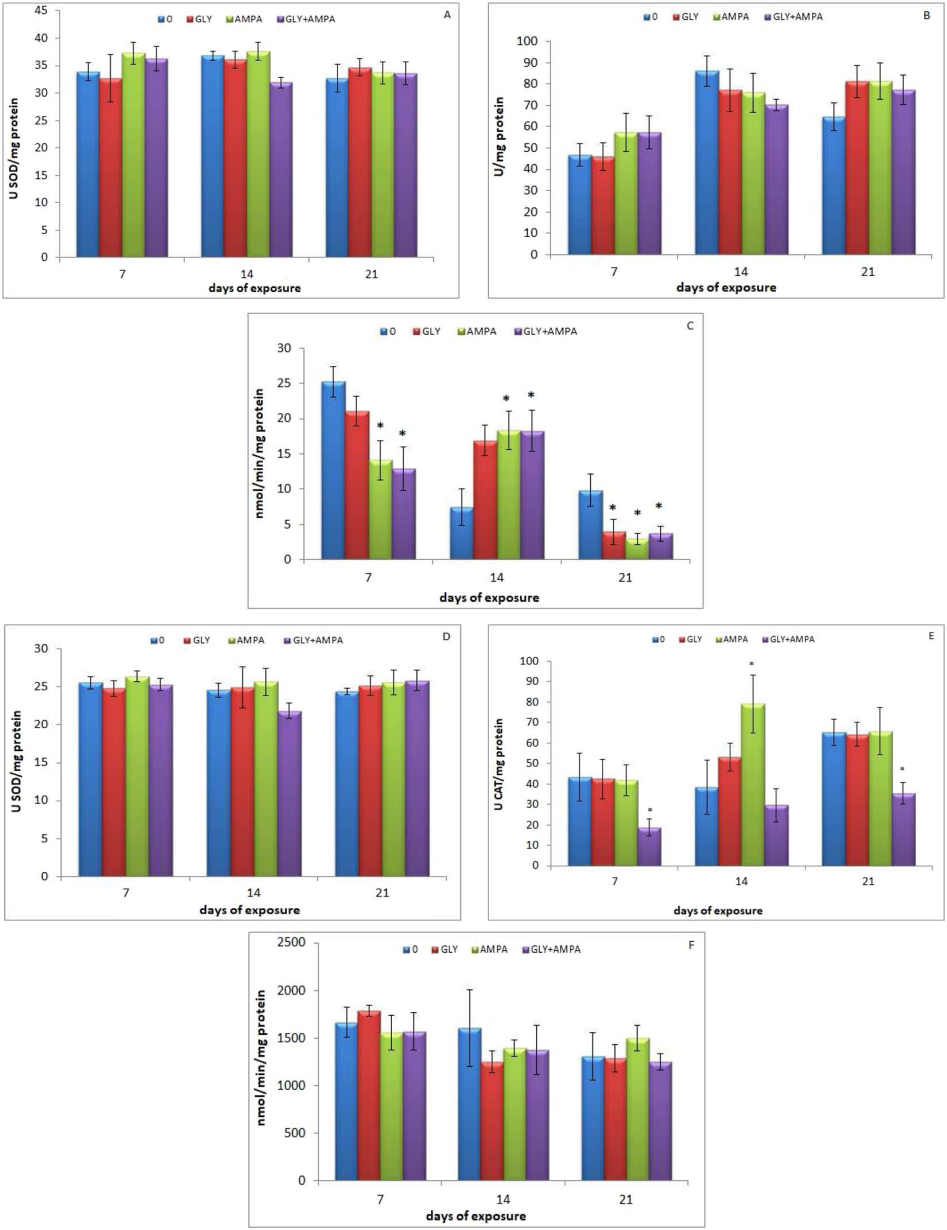
SOD and CAT activities, expressed as U/mg protein, and AChE activity, expressed as nmol/min/mg protein in gills (**A**–**C**), SOD and CAT activities, expressed as U/mg protein, and GST activity, expressed as nmol/min/mg protein in digestive gland (**D**–**F**) of *M*. *galloprovincialis* exposed to glyphosate (GLY), AMPA and GLY + AMPA. The values are mean + SD (n = 5). The asterisks indicate significant differences in comparison with controls: *p < 0.05.

Considering separately the results obtained in the two tissues, PERMANOVA analysis revealed significant effects of the variables “exposure time” (p < 0.001) and “treatment*time interaction” (p < 0.05) on gill biomarkers (Table 1). PERMANOVA analysis performed on individual biomarker results demonstrated no significant variations of SOD activity (Table 1; Fig. 2A), whereas a significant (p < 0.001) time-dependent effect on CAT and AChE activities was observed (Table 1; Fig. 2B,C). In addition, AChE resulted significantly (p < 0.05) influenced by “treatment*time interaction” variable (Table 1). As for pairwise comparisons, AChE activity was shown to decrease significantly (p < 0.05) in bivalves treated for 7 days with AMPA and the mixture, increased (p < 0.05) in those exposed for 14 days at the same experimental conditions, and decreased again (p < 0.05) in mussels exposed for 21 days to the three experimental concentrations tested, with respect to controls (Fig. 2C).

As for digestive gland parameters, a significant (p < 0.05) effect of the variable “treatment” (Table 1) was recorded. The three variables considered did not affect SOD activity during the study (Table 1; Fig. 2D). Conversely, “treatment” significantly (p < 0.01) affected CAT activity (Table 1; Fig. 2E), whereas “exposure time” influenced significantly (p < 0.01) GST activity (Table 1; Fig. 2F). In detail, a statistically significant (p < 0.05) decrease of CAT activity was observed in mussels exposed for 7 and 21 days to the mixture, whereas CAT activity increased significantly (p < 0.05) in those treated for 14 days with AMPA, compared to the related controls (Fig. 2E).

### CCA analysis

**Figure 3:**
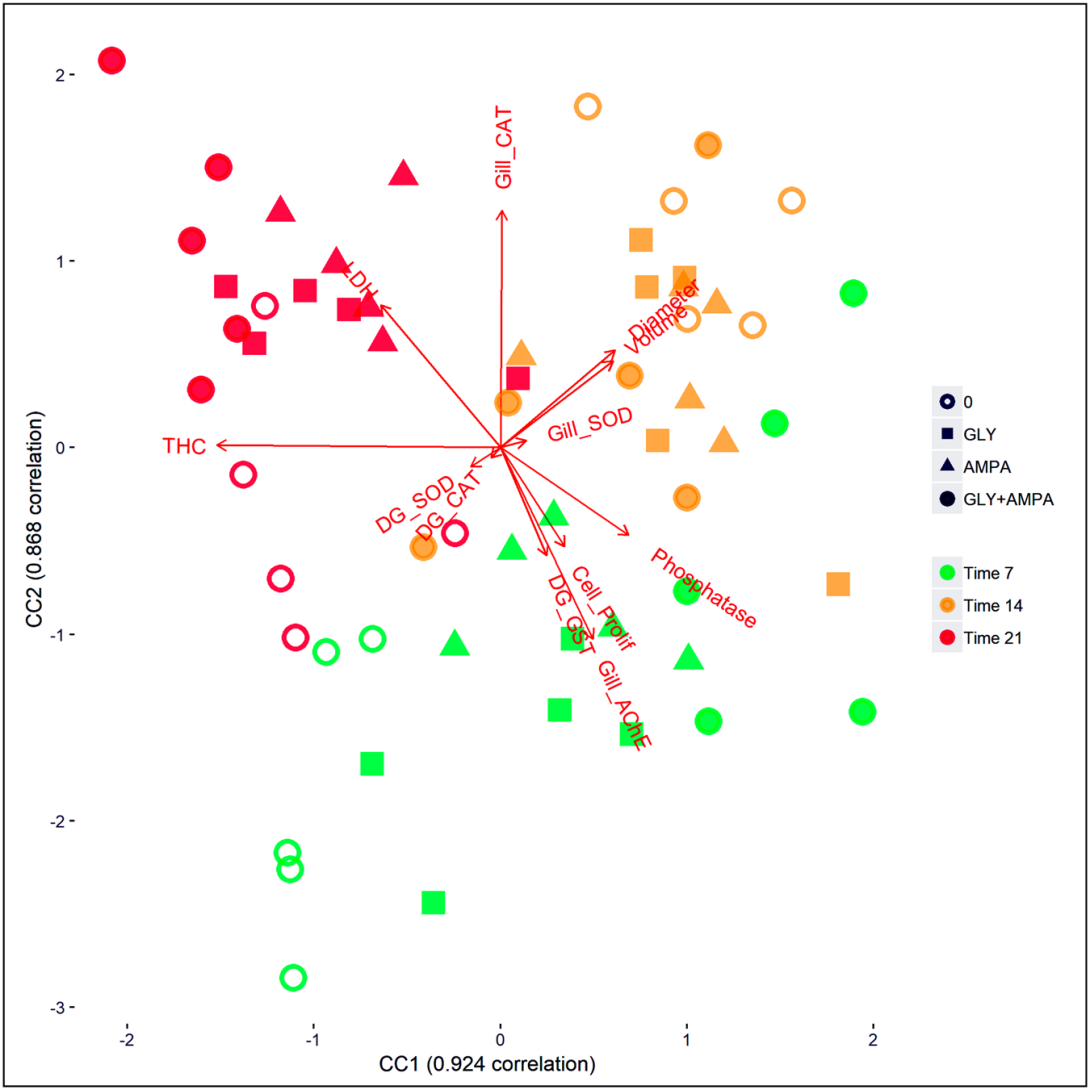
CCA analysis results. Both the biomarkers measured in mussels and the experimental conditions (treatment and exposure duration) were considered. T.7, T.14 and T.21 correspond to tissue sampling times, namely after 7, 14 and 21 days of exposure. 0, GLY, AMPA and GLY + AMPA are the compounds that were tested.

A plot of the first two components of the CCA analysis are depicted in the biplot in Fig. 3. The first principal correlations are 92% and 87% for the first and the second canonical correlation, respectively. The samples highlighted a marked influence of the experimental plan on the biomarkers. Moreover, a clear time-dependent pattern of variation can be highlighted, Time 1 (= 7 days, in the bottom part) being clearly separated from Times 2–3 (= 14 and 21 days, respectively; in the upper part). Samples from dose = 0 are well separated from other doses, especially at Time 7. Dose “0” and dose “GLY + AMPA” (= mixture) show the largest distance in all the times (most pronounced at Time 7).

## Discussion

In the current ecotoxicological studies it is crucial to evaluate the effects of contaminant mixtures in order to highlight the risk posed by differing substances occurring simultaneously in the environment^34^. In this study, a suite of biomarkers was measured in differing tissues from mussels to assess the impact of environmentally relevant concentrations of glyphosate and AMPA, alone or as a mixture.

### Haemocyte parameters

At the cellular level, our study demonstrated that glyphosate and AMPA (alone or in combination) affected THC values in mussels, even if with a different pattern of variation between 7 (decreasing trend) and 14 days (increasing trend) of exposure. Significant decreases in THC values were previously recorded in *M*. *galloprovincialis* exposed in two distinct experiments for 7 and 14 days to glyphosate^24^ and for 21 days to AMPA^25^. Interestingly, in the present study despite a different pattern of variation in THC values was recorded in bivalves treated for 7 and 14 days, it can be highlighted that exposure to the mixture of glyphosate and AMPA exacerbated the effects of the individual compounds. In addition, results obtained suggested that mussels exposed for 14 days were trying to compensate the initial (7 days) drastic reduction in THC, mainly in bivalves exposed to the mixture. It is commonly assumed that THC increases owing to either cell proliferation or mobilisation of haemocytes from peripheral tissues into the haemolymph, whereas THC decrease owing to cell death or the enhanced movement of haemocytes from the haemolymph into tissues^35,36^. In our study, no significant correlation between THC and haemocyte proliferation (Pearson’s correlation coefficient r = −0.11, p = 0.367) was recorded. Therefore, we speculated that the reduction recorded in bivalves following 7 days of exposure to all the experimental conditions tested was caused by increased haemocyte mobilisation from haemolymph into the peripheral tissues. Alternatively, it can be hypothesised that the significant increase in cell proliferation recorded in mussels exposed for 7 days was due to a strategy of animals to compensate - partially at least - the reduction in the number of circulating haemocytes. As for available data on the effects of glyphosate and AMPA on this important cell parameters, a recent study demonstrated significant reductions in THC values in the freshwater shrimp, *Macrobrachium nipponensis*, exposed for 24 h to 1.40, 2.80 and 5.60 mg/L glyphosate, and for 96 h to 5.60 mg/L^16^. In the crab *Eriocheir sinensis*, THC decreased significantly after 6 h of exposure to all glyphosate concentrations tested (4.4 to 98 mg/L), with highest reduction of 42.21% at 98 mg/L^17^. Conversely, 7 days of exposure to 10 mg/L of Roundup® (= 48% of glyphosate) increased significantly THC in the snail *Biomphalaria alexandrina*, both in *Schistosoma mansoni-*infected animals and healthy snails^37^. Overall, this study suggested that the mixture of glyphosate and AMPA affected markedly THC values in *M*. *galloprovincialis*, even if the pattern of variation of THC was different between mussels treated for 7 days and those treated for 74 days.

In this study, although slight increases in both diameter and volume of haemocytes were generally recorded in *M*. *galloprovincialis*, differences were not significant with respect the related controls. Similarly, no significant changes in haemocyte size were found in *Crassostrea gigas* following exposure to the pesticide metaldehyde^38^. Conversely, in our previous survey, glyphosate increased significantly cell volume in haemocytes from *M*. *galloprovincialis* exposed for 7 days to 1000 µg/L and 21 days to 10 µg/L^24^. As for AMPA, our previous study demonstrated that haemocyte diameter decreased significantly in bivalves treated for 14 days with 100 µg/L, whereas haemocyte volume was shown to increase significantly in animals exposed for 14 days to 1 and 10 µg/L of AMPA and decreased significantly at 100 µg/L, with respect to controls^25^. Results of our study suggested that the mixture of glyphosate and AMPA did not influence haemocyte size in *M*. *galloprovincialis*.

As stated above, in the present study haemocyte proliferation increased significantly in animals exposed for 7 days to both glyphosate and mixture, and in those treated for 14 days with AMPA. Conversely, cell proliferation decreased significantly in bivalves treated for 21 days with the mixture. We have previously demonstrated that exposure of *M*. *galloprovincialis* to the highest concentration of AMPA induced a significant increase in haemocyte proliferation throughout the study^25^. However, in this study no correlation between haemocyte proliferation and THC values was recorded. Consequently, we speculated that haemocyte proliferation increased in treated mussels to compensate - at least partially - for the reduction in THC, as already suggested for AMPA-exposed mussels^25^. Unfortunately, no further information about the effects of glyphosate and AMPA (alone or as a mixture) on cell proliferation is available in the literature, to the best of our knowledge at least.

LDH is a cytoplasmic enzyme that can be released into the extracellular fluids by damaged cells. Consequently, LDH assay can be used to detect signs of cell membrane destabilisation. Our study indicated that exposure to the mixture increased significantly LDH activity in CFH of mussels exposed for 21 days, suggesting a reduction in haemocyte membrane stability. In our previous studies, exposure to glyphosate was shown to increase significantly LDH activity in CFH from mussels exposed for 7 (at 10 and 1000 µg/L) and 14 days (at 10 µg/L)^24^, whereas exposure to AMPA increased significantly LDH activity in animals treated for 14 with 100 µg/L and for 21 days with the three concentrations tested^25^. Destabilisation of cell membrane was also recorded in other mollusc species exposed to Roundup®^39^, to the herbicides fomesafen^40^ and atrazine^41^. Overall, this study suggested that haemocyte membrane stability can be affected by the mixture of glyphosate and AMPA, similarly to what was observed previously by testing individual compounds.

Regarding lysosomal hydrolytic enzymes, this study demonstrated that exposure of mussels to the compounds tested did not alter significantly acid phosphatase activity. Non-linear results have previously been obtained after exposure of *M*. *galloprovincialis* to glyphosate alone, acid phosphatase activity decreasing significantly in HL of animals treated for 7 days with 1000 µg/L and for 21 days with 100 and 1000 µg/L, and increasing after exposure for 14 days to 1000 µg/L^24^. Following treatment of the crab *Eriocheir sinensis* with glyphosate, acid phosphatase activity was shown to decrease markedly in CFH from animals exposed for 6 h to 96 h to relatively high concentrations of the contaminant (e.g., 44 and 98 mg/L)^17^. On the basis of the results of this study, we can state that acid phosphatase is not a target of the mixture of glyphosate and AMPA.

### Gill and digestive gland enzyme activities

Of the enzyme activities measured in *M*. *galloprovincialis*, only gill AChE and digestive gland CAT resulted influenced significantly by the variables “treatment” and “treatment*time interaction”, respectively. Regarding antioxidant enzymes, exposure to the mixture of glyphosate and AMPA was shown to decrease CAT activity, in particular in digestive gland of mussels treated for 7 and 21 days. Conversely, a significant reduction in soft tissue SOD activity (but not in CAT activity) was observed in the golden mussel *L*. *fortunei* following exposure for 26 days to glyphosate (1, 3 and 6 mg/L)^42^. In a recent study conducted by the same authors on the same mussel species demonstrated that glyphosate did not affect significantly SOD and CAT activities after 28 days of dietary exposure with the microalgae *Scenedesmus vacuolatus* previously treated with a commercial formulation of glyphosate (6 mg/L, as active principle)^22^. The authors suggested that differences in the results of the two studies^22,42^ could be due to exposure modality of mussels (glyphosate dose and availability in experiments with treated microalgae could be lower than those in waterborne exposure). In the clam *Corbicula fluminea*, exposure for 96 h to 2 and 10 mg/L of Roundup® caused a significant increase in SOD activity in gills and a significant decrease in CAT activity of digestive gland from animals treated with the highest concentration tested^21^. In the freshwater shrimp *M*. *nipponensis*, SOD and CAT activities and total antioxidant capacity was shown to decrease in a dose- and time-dependent manner in serum of animals exposed for 96 h to high concentrations (0.70, 1.40, 2.80 and 5.60 mg/L) of Roundup®^16^. Overall, the results obtained in this study suggested that exposure to the mixture of glyphosate and AMPA affects antioxidant enzyme activity in *M*. *galloprovincialis*, as for digestive gland and CAT activity at least.

AChE activity measurement is a useful biomarker to assess the effects of neurotoxic compounds - organophosphate compounds in particular - in marine organisms^43,44^. Results obtained in this study demonstrated that the exposure to both AMPA and the mixture (and to glyphosate at 21 days) affected significantly AChE activity in mussels. Interestingly, a biphasic response of animals to the contaminants was recorded: AChE activity decreased after 7 days of exposure to AMPA and the mixture, increased after 14 days at the same experimental conditions, and decreased again after 21 days at all the doses tested. It can be hypothesised that AChE activity increased in 14 days-exposed mussels to balance the initial reduction recorded at 7 days, whereas enzyme inhibition recorded after 21 days suggested inability of mussels to counteract exposure effects. In *M*. *nipponensis*, exposure to Roundup® decreased significantly AChE activity in serum of shrimps exposed for 24 h to 96 h^16^. In an *in vitro* study, glyphosate (0.075 to 15 mM) was shown to inhibit cholinesterase activity in gills and muscle from the mussel *Perna perna* and in brain and muscle from the fish *Danio rerio* and *Jenynsia multidentata*^45^. Conversely, no significant alterations in AChE activity were recorded in *L*. *fortunei* following direct exposure to glyphosate^42^ or via glyphosate-contaminated food (microalgae)^22^. Overall, results of our study suggested that the mixture of glyphosate and AMPA affected significantly AChE activity in mussels.

GST is involved in phase II of xenobiotic detoxification process^46^. The resulting products are more hydrophilic and more easily excreted^47^. Generally, increased GST activity indicates high capability of tissues/cells to remove xenobiotics. In the present study, no significant variations in GST activity were observed in digestive gland of bivalves exposed to glyphosate, AMPA and their mixture. Similarly, no significant alterations in GST activity were found in gills and digestive gland of *C*. *fluminea* following exposure for 96 h to 2 and 10 mg/L of Roundup®^21^. Conversely, a significant increase (72%) in the GST activity was observed in *L*. *fortunei* fed for 28 days with the glyphosate-treated microalgae *Scenedesmus vacuolatus*^22^. The exposure for 42 days to the glyphosate-containing formulation Roundup Max® (0.2 and 2 mg/L) induced a significant decrease in GST activity in the liver of the fish *Cnesterodon decemmaculatus*, whereas no significant changes were recorded in brain, gills and muscle^48^.

Summarising, our study demonstrated that exposure to a mixture of realistic doses of glyphosate and AMPA influences cell and tissue biomarkers in the mussel *M*. *galloprovincialis*. In particular, inhibition of AChE activity in mussel gills is noteworthy. Although further studies are necessary to elucidate better the mode of action of the contaminants tested (alone or in combination), results reported here suggest a potential ecotoxicological risk for bivalve molluscs.
